# Alterations in the aqueous humor proteome in patients with a glaucoma shunt device

**Published:** 2011-07-14

**Authors:** Arundhati Anshu, Marianne O. Price, Matthew R. Richardson, Zaneer M. Segu, Xianyin Lai, Mervin C. Yoder, Francis W. Price

**Affiliations:** 1 Price Vision Group, Indianapolis, IN; 2 Cornea Research Foundation of America, Indianapolis, IN; 3 Department of Pediatrics, Indiana University School of Medicine, Indianapolis, IN; 4 Department of Chemistry, Indiana University, Bloomington, IN; 5 Department of Cellular and Integrative Physiology, Indiana University School of Medicine, Indianapolis, IN

## Abstract

**Purpose:**

To investigate whether implantation of a glaucoma shunt device leads to inappropriate accumulation of plasma derived proteins in the aqueous humor.

**Methods:**

Aqueous humor samples were collected from 11 patients (study group) with a glaucoma shunt device undergoing either cataract surgery or a corneal transplant and 11 patients (control) with senile cataract undergoing routine cataract extraction. Of the study group, 9 had an Ahmed valve implant and 2 eyes had a Baerveldt implant. Tryptic digests of the mixture of proteins in aqueous humor (AH) were analyzed using Liquid Chromatography/Mass Spectrometry (LC-MS/MS). Proteins were identified with high confidence using stringent criteria and compared quantitatively using a label-free platform (IdentiQuantXL^TM^).

**Results:**

We identified 135 proteins in the albumin-depleted fraction in both the study and control group AH. Using stringent criteria, 13 proteins were detected at a significantly higher level compared to controls. These proteins are known to play a role in oxidative stress, apoptosis, inflammation and/or immunity and include gelsolin (p=0.00005), plasminogen (p=0.00009), angiotensinogen (p=0.0001), apolipoprotein A-II (p=0.0002), beta-2-microglobulin (p=0.0002), dickkopf-3 (DKK-3; p=0.0002), pigment epithelium-derived factor (p=0.0002), RIG-like 7–1 (p=0.0002), afamin (p=0.0003), fibronectin 1 (FN1; p=0.0003), apolipoprotein A-I (p=0.0004), activated complement C4 protein (C4a; p=0.0004) and prothrombin (p=0.0004). Many of the identified proteins were novel proteins that have not been associated with glaucoma in prior studies. All but C4a (complement C4 is a plasma protein but not in an activated form) are known plasma proteins and the elevated levels of these proteins in the aqueous humor suggests a breach in the blood-aqueous barrier with passive influx into the anterior chamber of the eye.

**Conclusions:**

The presence of these proteins in the aqueous humor suggests that glaucoma shunt device causes either a breach in blood-aqueous barrier or chronic trauma, increasing influx of oxidative, apoptotic and inflammatory proteins that could potentially cause corneal endothelial damage.

## Introduction

Glaucoma is an optic neuropathy characterized by progressive loss of retinal ganglion cells that lead to structural changes at the optic nerve head and functional visual loss. It is often, but not always, associated with increased intra-ocular pressure (IOP). According to the World Health Organization, it is the second leading cause of blindness in the world and accounts for 9%–12% of cases of blindness in the US. Management strategies include medical therapy usually in the form of topical anti-glaucoma medications to lower IOP. If IOP is resistant to medical therapy and/or there is progressive optic nerve damage, surgery is considered, usually in the form of trabeculectomy or a glaucoma shunt device.

Glaucoma has long been recognized as an important factor influencing corneal graft survival [1-5]. In our published series of over 4,000 full thickness penetrating keratoplasties, we identified pre-existing glaucoma as a risk factor for graft failure and other authors have reported similar outcomes [2,6,7]. High intra-ocular pressure (IOP) is not only detrimental to optic nerve function, it can also lead to corneal endothelial cell attrition. Glaucoma filtration surgery, although essential for preservation of visual function, has also been known to affect corneal graft survival adversely with several series citing poor longer-term graft survival especially in eyes with a glaucoma shunt device [8-10]. More recently, in eyes undergoing endothelial keratoplasty, glaucoma filtration surgery also had a significantly adverse effect on graft survival [11].

The mechanisms of corneal endothelial damage in eyes with a glaucoma shunt device are not fully understood. Glaucoma shunt devices can damage the corneal endothelium by mechanical means or by permitting retrograde entrance of inflammatory cells into the anterior chamber [12-15]. In addition, glaucoma shunt devices disrupt the blood–aqueous barrier and this could further increase influx of inflammatory mediators that could potentially cause corneal endothelial damage.

Proteomics is one of the emerging techniques for biomarker discovery. Aqueous humor (AH) is the biologic fluid in the eye that has the task of protecting and supplying nutrition to the cornea, lens and trabecular meshwork (TM). A balance between production and drainage of AH is critical to maintaining normal IOP. The protein composition of AH has been shown to change dramatically in various ocular conditions such as corneal graft rejection [16], myopia [17], corneal dystrophies [18-20], and glaucoma [21-24]. Although the exact pathogenesis of glaucoma remains unclear, it is likely that alterations in the AH protein composition trigger signaling molecules that could modify the TM, increasing resistance to outflow and hence glaucoma [25,26].

With this background in mind, we decided to explore the AH proteomics in eyes with a pre-existing glaucoma shunt device to characterize the proteins that could potentially serve as biomarkers for not only glaucoma but also for corneal endothelial damage. The results of this study could potentially influence therapeutic strategies designed to improve longer-term graft survival in these high-risk eyes.

## Methods

### Sample collection

Patients were selected and samples collected as previously described [27]. Briefly, study subjects were either patients scheduled to undergo routine cataract surgery (controls) or patients with previous glaucoma shunt device scheduled to undergo corneal transplant or cataract surgery at a tertiary referral center, Price Vision Group (Indianapolis, IN). Exclusion criteria were as follows: history of conjunctivitis or any ocular infection within the previous 3 months and ongoing intraocular inflammation. An independent review board (IRB) approved the study and all subjects signed a written Informed Consent document. Before surgery, the patient's eye was anesthetized topically with proparacaine. A stab incision was made in the peripheral cornea, and 0.1 to 0.2 ml of anterior chamber fluid was aspirated using a 30-gauge needle. AH samples were stored frozen in liquid nitrogen until analysis. Any sample suspected of being contaminated with blood or iris pigment was discarded. Samples from 22 subjects were analyzed (11 cataract patients and 11 patients with glaucoma shunt device) as shown in Table 1.

**Table 1:** Clinical data on study patients and normal controls.

**Age**	**Sex**	**Type of surgery during aqueous humor tap**
**Study patients**		
69	Female	Cataract
56	Male	Corneal transplant
31	Male	Cataract
56	Male	Corneal transplant
23	Male	Corneal transplant
74	Male	Corneal transplant
45	Male	Cataract
90	Female	Corneal transplant
49	Male	Corneal transplant
89	Male	Corneal transplant
71	Female	Repeat glaucoma shunt
**Normal controls**		
52	Male	Cataract
73	Male	Cataract
76	Female	Cataract
72	Male	Cataract
70	Male	Cataract
73	Male	Cataract
63	Female	Cataract
43	Female	Cataract
60	Female	Cataract
66	Female	Cataract
56	Female	Cataract

### Materials

Acetonitrile and ammonium bicarbonate were purchased from Fisher Scientific (Fair Lawn, NJ). Dithiothreitol (DTT) and iodoacetamide (IAA) were obtained from Bio-Rad Laboratories (Hercules, CA). Trypsin was purchased from Promega (Madison, WI). ProteoPrep immunoaffinity depletion kit was purchased from Sigma (St. Louis, MO). The following sample preparation and mass spectrometric analyses were performed at MetaCyt Biochemical Analysis Center (Bloomington, IN).

### Depletion and protein assay

Depletion of albumin and IgG was performed using the ProteoPrep immunoaffinity depletion kit (Sigma) as described in instruction manual with some modification. As the depletion kit is designed for plasma samples and the protein content in AH is significantly lower, preliminary studies were performed to develop a protocol for optimal AH depletion, which resulted in enhanced protein identification (data not shown). Briefly, an estimation of material to be used to deplete albumin and IgG from AH was made using a BCA protein assay and quantification of albumin and IgG in AH samples relative to plasma assuming total protein content of 80 µg/µl and 75% albumin and IgG in plasma. The estimated amount of material by weight was measured from the ProteoPrep immunoaffinity column and transferred to an empty spin column, and depletion was performed as described in the instruction manual.

### Trypsin digestion

Protein samples were subjected to tryptic digestion before analysis as follows: after thermal denaturation at 95 °C for 5 min, samples were reduced through the addition of DTT to a final concentration of 5 mM and incubated at 60 °C for 45 min. Alkylation was then followed by an addition of IAA to a final concentration of 20 mM for 45 min in the dark at room temperature. A second aliquot of DTT was then added, increasing the final concentration of DTT to about 10 mM. The samples were then incubated at room temperature for 30 min to quench the alkylation reaction. Next, trypsin was added (1:30 w/w) and microwave-assisted enzymatic digestion was performed at 45 °C for 15 min at the power of 50 W. Finally enzymatic digestion was quenched through the addition of 0.5 µl of neat formic acid.

### Instrumentation

Liquid chromatography tandem mass spectrometry (LC-MS/MS) analyses of the tryptic digests were performed using a Dionex 3000 Ultimate nano-LC system (Dionex, Sunnyvale, CA) interfaced to an LTQ Orbitrap hybrid mass spectrometer (Thermo Scientific, San Jose, CA). Prior to separation, a 4-µl aliquot of trypsin digestion (1 µg protein equivalent) was loaded onto a PepMap300 C18 cartridge (5 µm, 300 Å; Dionex) and eluted through the analytical column (150 mm×100 µm i.d, 200 Å pores) packed with C18 magic (Michrom Bioresources, Auburn, CA). Peptides originating from protein tryptic digests were separated using a reversed-phase gradient from 10%–55% B, 99.9% acetonitrile with 0.1% formic acid over 50 min for proteins isolated from the aqueous humor, at 500 nl/min flow rate and passed through an ADVANCE ionization source (Michrom Bioresources). The mass spectrometer was operated in an automated data-dependent mode that was switching between MS scan and CID-MS. In this mode, eluted LC products undergo an initial full-spectrum MS scan from *m*/*z* 300 to 2,000 in the Orbitrap at 15,000 mass resolutions, and subsequently CID-MS (at 35% normalized collision energy) was performed in the ion trap. The precursor ion was isolated using the data-dependent acquisition mode with a 2 *m*/*z* isolation width to select automatically and sequentially five most intense ions (starting with the most intense) from the survey scan. The total cycle (6 scans) was continuously repeated for the entire LC-MS run under data-dependent conditions with dynamic exclusion set to 60 s. Performing MS scanning in the Orbitrap offers high mass accuracy and accurate charge state assignment of the selected precursor ions.

### Protein identification and label-free quantification

#### Peptide and protein identification

The acquired data were searched against the International Protein Index (IPI) human database (ipi.HUMAN.v3.69.fasta) using SEQUEST (v. Twenty-eight rev. 12) algorithms in Bioworks (v. 3.3). General parameters were set to: peptide tolerance 2.0 amu, fragment ion tolerance 1.0 amu, enzyme limits set as “fully enzymatic - cleaves at both ends,” and missed cleavage sites set at 2. The searched peptides and proteins were validated by PeptideProphet [28] and ProteinProphet [29] in the Trans-Proteomic Pipeline (TPP, v. 3.3.0). Only proteins with probability ≥0.9000 and peptides with probability ≥0.8000 were reported.

#### Protein quantification

Protein quantification was performed using an in-house software package, IdentiQuantXL^TM^. The retention time of peptide for its intensity extraction was performed with an experiment-based algorithm RetentionTimeXL^TM^. The intensity of validated peptide was extracted and the protein quantification was calculated from peptide intensity.

### Statistical analysis

The relative quantity of each protein was determined in each individual AH sample, and the results for the two groups were compared using the Student’s *t*-test. A p-value <0.05 was considered to be statistically significant but then post-hoc adjustment of the p-value was performed using Holm-Sidak test to correct for multiple comparisons.

## Results

We performed label-free quantitative mass spectrometry on AH samples derived from patients with and without prior glaucoma shunt surgery. AH samples were depleted of interfering abundant proteins such as albumin before LC-MSMS was used for protein identification and quantification. We identified 135 proteins in the albumin-depleted fraction (Table 2) with high confidence in the study as well as control group AH. After using stringent detection criteria, the AH in eyes with a prior glaucoma shunt device showed significantly increased levels of 13 proteins as shown in bold in Table 2. These proteins were pro-inflammatory (plasminogen [p=0.00009], angiotensinogen [p=0.0001], prothrombin [p=0.0004] and C4a protein [p=0.0004]); anti-oxidative/anti-apoptotic (gelsolin [p=0.00005], afamin [p=0.0003] pigment epithelium-derived factor [PEDF; p=0.0002], and dickkopf-3 [DKK-3; p=0.0002]); anti-inflammatory (apolipoprotein A-I [apo A-1; p=0.0004], and apolipoprotein A-II [apo A-II; p=0.0002]); or other roles (fibronectin 1 [FN1; p=0.0003], RIG-like 7–1 [p=0.0002], and beta-2-microglobulin [p=0.0002]). The percent of protein sequence covered by the peptides identified with high confidence is listed for each protein along with the number of unique sequences as well as the fold-change compared to the protein level in control patients.

**Table 2:** Proteins identified in the albumin-depleted fraction of aqueous humor in patients with glaucoma shunt device using Liquid Chromatography/Mass Spectrometry (LC-MS/MS).

**Protein ID**	**Protein name**	**Protein coverage (%)**	**Number of unique sequences**	**CV (%) normal**	**CV (%) shunt**	**Fold change in shunt patients**	**p-value (shunt versus normal)**	**Plasma protein**
**IPI00026314**	**Gelsolin**	**5.12**	**2**	**97.38**	**42.91**	**4.7**	**0.000050**	**Yes**
**IPI00019580**	**Plasminogen**	**21.98**	**10**	**66.75**	**47.85**	**6.2**	**0.000090**	**Yes**
**IPI00032220**	**Angiotensinogen**	**16.49**	**5**	**50.43**	**46.49**	**4.4**	**0.000100**	**Yes**
**IPI00021854**	**Apolipoprotein A-II**	**64**	**6**	**71.48**	**58.76**	**18.5**	**0.000200**	**Yes**
**IPI00004656**	**Beta-2-microglobulin**	**18.49**	**1**	**33.81**	**49.13**	**5.7**	**0.000200**	**Yes**
**IPI00383937**	**cDNA FLJ33633 fis, clone BRAMY2022786, highly similar to Homo sapiens dickkopf-3 (DKK-3) mRNA**	**30.7**	**4**	**43.93**	**45.25**	**4**	**0.000200**	**Yes**
**IPI00002714**	**cDNA FLJ52545, highly similar to Dickkopf-related protein 3**	**18.13**	**4**	**43.93**	**45.25**	**4**	**0.000200**	**Yes**
**IPI00006114**	**Pigment epithelium-derived factor**	**38.28**	**12**	**54.02**	**48.22**	**4.6**	**0.000200**	**Yes**
**IPI00386812**	**RIG-like 7–1**	**23.39**	**2**	**35.69**	**52.57**	**7.2**	**0.000200**	**Yes**
**IPI00019943**	**Afamin**	**13.86**	**5**	**53.94**	**45.3**	**3.3**	**0.000300**	**Yes**
**IPI00922213**	**cDNA FLJ53292, highly similar to Homo sapiens fibronectin 1 (FN1), transcript variant 5, mRNA**	**2.17**	**1**	**53.67**	**53.43**	**6.2**	**0.000300**	**Yes**
**IPI00021841**	**Apolipoprotein A-I**	**54.31**	**17**	**49.82**	**55.18**	**6.4**	**0.000400**	**Yes**
**IPI00889723**	**C4A protein**	**18.43**	**18**	**60.51**	**50.69**	**4.1**	**0.000400**	**No**
**IPI00019568**	**Prothrombin (Fragment)**	**18.81**	**7**	**59.4**	**43.88**	**2.9**	**0.000400**	**Yes**
**IPI00937598**	**similar to C4A protein isoform 2**	**15.9**	**15**	**59.85**	**45.91**	**3.3**	**0.000400**	**No**
IPI00017601	Ceruloplasmin	32.68	24	51.22	52.5	4.5	0.000500	
IPI00022488	Hemopexin	53.9	17	73.75	56.72	6.2	0.000500	
IPI00215894	Kininogen-1, Isoform LMW	26.93	8	49.09	47.4	3.2	0.000600	
IPI00789376	KNG1 protein	34.36	7	48.72	47.54	3.1	0.000700	
IPI00163207	N-acetylmuramoyl-L-alanine amidase	4.69	2	64.87	53.66	4.2	0.000700	
IPI00909649	Protein	49.53	3	52.92	61.2	7.2	0.000800	
IPI00893864	Complement factor B	6.45	2	24.5	55.96	4.4	0.000900	
IPI00022420	Retinol-binding protein 4	33.33	4	63.72	53.93	3.9	0.000900	
IPI00022463	Serotransferrin	67.62	50	66.88	50.61	3.4	0.000900	
IPI00032179	Antithrombin-III	43.1	14	62.13	59.34	4.7	0.001000	
IPI00922058	cDNA FLJ59472, highly similar to Tripeptidyl-peptidase 1	7.8	1	28.97	41.42	2.3	0.001000	
IPI00783987	Complement C3 (Fragment)	29.95	34	57.03	54.67	3.6	0.001000	
IPI00892547	Complement component 4A	20.3	20	59.62	59.21	5	0.001000	
IPI00027482	Corticosteroid-binding globulin	10.12	2	45.96	61.64	5.4	0.001000	
IPI00556287	Putative uncharacterized protein	17.99	2	34.29	56.42	4.3	0.001000	
IPI00924948	Putative uncharacterized protein AZGP1	26.87	4	66.28	55.65	4	0.001000	
IPI00643525	Putative uncharacterized protein C4A	21.33	21	58.55	59.2	5.2	0.001000	
IPI00739237	similar to complement component 3	4.87	1	42.7	58.4	4.7	0.001000	
IPI00935601	similar to complement component 4B (Childo blood group), partial	2.85	1	92.62	61.48	6	0.001000	
IPI00298971	Vitronectin	13.6	4	56.8	59.77	5.1	0.001000	
IPI00020091	Alpha-1-acid glycoprotein 2	39.8	8	74.23	60.87	4.2	0.002000	
IPI00022895	Alpha-1B-glycoprotein	35.96	11	64.92	68.72	6.5	0.002000	
IPI00166729	alpha-2-glycoprotein 1, zinc precursor	40.6	9	55.39	51.01	2.9	0.002000	
IPI00478003	Alpha-2-macroglobulin	11.47	11	56.79	54.7	3.3	0.002000	
IPI00218748	Beta-crystallin B2	19.51	4	95.69	65.53	5.7	0.002000	
IPI00019591	cDNA FLJ55673, highly similar to Complement factor B	5.45	4	44.43	58.6	4.2	0.002000	
IPI00032258	Complement C4-A	20.07	20	58	62.3	5	0.002000	
IPI00654875	Complement C4-B	18.18	18	59.41	55.54	3.5	0.002000	
IPI00218192	Inter-alpha-trypsin inhibitor heavy chain H4	7.33	4	66.74	67.97	7.6	0.002000	
IPI00032328	Kininogen-1, Isoform HMW	15.84	7	50.67	51.24	2.9	0.002000	
IPI00384938	Putative uncharacterized protein DKFZp686N02209	29.88	9	68.21	57.76	3.6	0.002000	
IPI00014048	RNase pancreatic	13.46	1	52.72	61.62	4.8	0.002000	
IPI00553177	Alpha-1-antitrypsin	66.27	30	75.69	68.19	5.9	0.003000	
IPI00947307	cDNA FLJ58075, highly similar to Ceruloplasmin	15.75	9	75.54	71.62	7.1	0.003000	
IPI00892604	Complement component C4B (Childo blood group) 2	26.09	27	57.45	64.62	4.5	0.003000	
IPI00013179	Prostaglandin-H2 D-isomerase	32.63	3	59.4	52.56	2.7	0.003000	
IPI00930124	Putative uncharacterized protein DKFZp686C11235	24.1	7	68.59	60.18	3.6	0.003000	
IPI00947496	124 kDa protein	6.46	5	72.67	68.22	4.4	0.004000	
IPI00939333	86 kDa protein	11.13	5	57.33	57.59	3	0.004000	
IPI00477597	Haptoglobin-related protein	20.98	7	48.71	67.78	4.6	0.004000	
IPI00745872	Serum albumin	56.98	31	62.39	65.64	4	0.004000	
IPI00796888	26 kDa protein	7.76	1	28.92	68.46	4.1	0.005000	
IPI00942927	cDNA FLJ57339, highly similar to Complement C3	5.28	3	74.79	75.8	6.2	0.005000	
IPI00796830	13 kDa protein	10.53	1	63.45	55.98	2.6	0.006000	
IPI00304273	Apolipoprotein A-IV	37.37	14	62.49	69.08	4.1	0.006000	
IPI00924859	kininogen-1, Isoform 3	26.6	7	53.65	61.14	3	0.006000	
IPI00555812	Vitamin D-binding protein	42.62	17	62.88	76.48	5.7	0.006000	
IPI00022426	Protein AMBP	17.61	4	78.76	46.73	2.4	0.007000	
IPI00550991	cDNA FLJ35730 fis, clone TESTI2003131, highly similar to ALPHA-1-ANTICHYMOTRYPSIN	43.53	16	51.65	68.56	3.6	0.008000	
IPI00908876	cDNA FLJ50830, highly similar to Serum albumin	7.27	2	126.5	89.61	13	0.009000	
IPI00879709	Complement component 6 precursor	2.65	1	137.14	82.89	6.9	0.009000	
IPI00884251	ENV polyprotein (coat polyprotein) family protein	5.29	1	101.32	85.96	8.3	0.009000	
IPI00291866	Plasma protease C1 inhibitor	20.6	7	50.85	68	3.3	0.009000	
IPI00022434	Putative uncharacterized protein ALB	33.17	15	84.91	83.54	7	0.009000	
IPI00877967	Putative uncharacterized protein F2	16.67	3	38.9	70.48	3.7	0.009000	
IPI00298828	Beta-2-glycoprotein 1	32.75	8	50.08	58.28	2.4	0.010000	
IPI00794184	cDNA FLJ37971 fis, clone CTONG2009958, highly similar to CERULOPLASMIN	5.2	2	43.15	54.38	2.2	0.010000	
IPI00910625	cDNA FLJ51265, moderately similar to Beta-2-glycoprotein 1	31.75	6	57.7	53.85	2.3	0.010000	
IPI00291262	Clusterin	21.83	8	64.87	81.71	4.9	0.010000	
IPI00019690	GRAM domain-containing protein 4	1.56	1	42.31	68.53	3.1	0.010000	
IPI00887739	similar to complement component C3, partial	3.01	2	99.17	85.98	6.6	0.010000	
IPI00556036	16 kDa protein	9.35	1	46.68	101.59	22.4	0.020000	
IPI00940791	20 kDa protein	26.49	4	68.87	66.04	2.5	0.020000	
IPI00022429	Alpha-1-acid glycoprotein 1	41.29	10	92.7	78.72	4	0.020000	
IPI00022395	Complement component C9	6.26	3	83.12	58.44	2.4	0.020000	
IPI00291867	Complement factor I	8.92	4	66.9	89.51	5.8	0.020000	
IPI00022371	Histidine-rich glycoprotein	18.48	6	52.49	65.69	2.7	0.020000	
IPI00016915	Insulin-like growth factor-binding protein 7	4.61	1	43.16	87.43	5.3	0.020000	
IPI00830047	Putative uncharacterized protein ENSP00000374858 (Fragment)	14.15	1	86.52	93.61	6.7	0.020000	
IPI00736860	Putative uncharacterized protein ENSP00000374988 (Fragment)	5.76	1	55.66	81.96	3.7	0.020000	
IPI00942787	42 kDa protein	34.82	13	61.83	91.49	4.4	0.030000	
IPI00922262	cDNA FLJ56822, highly similar to Alpha-2-HS-glycoprotein	6.94	2	82.08	90.01	4.1	0.030000	
IPI00641737	Haptoglobin	37.86	14	60.67	91.2	4.3	0.030000	
IPI00305461	Inter-alpha-trypsin inhibitor heavy chain H2	3.59	2	67.05	67.19	2.4	0.030000	
IPI00019038	Lysozyme C	35.14	3	99.3	100.08	6.8	0.030000	
IPI00855916	Transthyretin	55.43	12	56.66	51.02	1.9	0.030000	
IPI00910636	cDNA FLJ53848, highly similar to Inter-alpha-trypsin inhibitor heavy chain H2	2.76	1	105.83	80.27	3.2	0.040000	
IPI00032293	Cystatin-C	11.64	2	37.2	65.84	2.2	0.040000	
IPI00426060	Putative uncharacterized protein DKFZp686J11235 (Fragment)	24.11	8	50.51	70.47	2.4	0.040000	
IPI00218413	Biotinidase	3.13	1	152.06	59.58	2.5	0.050000	
IPI00296165	cDNA FLJ54471, highly similar to Complement C1r subcomponent	1.95	1	38.2	100.53	4.6	0.050000	
IPI00022418	Fibronectin	1.63	2	49.85	82.97	2.8	0.050000	
IPI00478493	Haptoglobin isoform 2 preproprotein	34.01	10	64.09	96.65	4.2	0.050000	
IPI00431645	HP protein	25.62	6	72.49	101.65	4.9	0.050000	
IPI00910432	cDNA FLJ57921, highly similar to Apolipoprotein D	10.94	1	32.12	115.55	6.7	0.060000	
IPI00156171	Ectonucleotide pyrophosphatase/phosphodiesterase family member 2	4.06	2	154.37	118.59	9.6	0.060000	
IPI00026199	Glutathione peroxidase 3	29.2	6	62.58	52.9	1.9	0.060000	
IPI00292150	Latent-transforming growth factor beta-binding protein 2	1.04	1	35.63	129.41	22.1	0.060000	
IPI00002678	Opticin	14.46	2	44.87	57.55	1.8	0.060000	
IPI00852577	HCG2040025	33.02	2	79.85	92.66	3.2	0.070000	
IPI00021000	Osteopontin	10.19	2	73.71	115.05	6.2	0.070000	
IPI00514159	Inter-alpha (Globulin) inhibitor H2	8.02	1	61.82	59.15	1.8	0.090000	
IPI00029863	55 kDa protein (Alpha-2-antiplasmin precursor)	8.69	2	55.95	78.97	−1.8	0.100000	
IPI00002147	Chitinase-3-like protein 1	9.92	2	56.83	128.11	4.3	0.100000	
IPI00009650	Lipocalin-1	6.25	1	66.59	61.11	1.8	0.100000	
IPI00844156	SERPINC1 protein	3.86	1	77.33	80.74	2.3	0.100000	
IPI00383164	SNC66 protein	19.72	7	64.5	79.48	2.1	0.100000	
IPI00794403	23 kDa protein	17.82	3	80.99	159.25	9.8	0.200000	
IPI00922298	cDNA FLJ51445, highly similar to AMBP protein	7.04	1	64.26	81.95	2	0.200000	
IPI00022431	cDNA FLJ55606, highly similar to Alpha-2-HS-glycoprotein	13.39	4	49.95	144.5	5.1	0.200000	
IPI00029739	Complement factor H	1.46	1	48.36	116.78	2.6	0.200000	
IPI00021891	Fibrinogen gamma chain	7.51	2	86.57	136.01	3.5	0.200000	
IPI00292530	Inter-alpha-trypsin inhibitor heavy chain H1	2.09	1	66.21	40.03	−1.7	0.200000	
IPI00020986	Lumican	16.86	4	72.95	148.83	7.5	0.200000	
IPI00514285	Prostaglandin D2 synthase 21 kDa	14.73	2	62.05	70.38	1.7	0.200000	
IPI00658053	Putative uncharacterized protein CTSD	8.54	1	46.63	73.25	1.7	0.200000	
IPI00877925	Putative uncharacterized protein SERPINF2	6.44	1	74.76	66.92	−1.5	0.200000	
IPI00218732	Serum paraoxonase/arylesterase 1	6.2	1	41.72	55.4	1.5	0.200000	
IPI00006662	Apolipoprotein D	20.11	3	201.07	113.63	2.7	0.300000	
IPI00165972	Complement factor D preproprotein	15	2	63.48	59	1.5	0.300000	
IPI00930442	Putative uncharacterized protein DKFZp686M24218	5.67	1	99.15	71.25	1.7	0.300000	
IPI00935408	CFI protein	4.24	1	81.47	78.69	1.3	0.400000	
IPI00022417	Leucine-rich alpha-2-glycoprotein	29.97	5	59.22	42.95	1.2	0.400000	
IPI00334282	Protein FAM3C	7.05	1	38.27	61.04	−1.1	0.500000	
IPI00012503	Proactivator polypeptide	2.86	1	262.28	79.65	1.8	0.600000	
IPI00807428	Putative uncharacterized protein	22.98	3	168.41	135.85	2.1	0.600000	
IPI00011229	Cathepsin D	8.5	2	106.86	100.28	−1.5	0.700000	
IPI00423460	Putative uncharacterized protein DKFZp686G21220 (Fragment)	9.88	3	104.19	116.55	1.6	0.700000	
IPI00022337	Retinol-binding protein 3	5.93	3	71.53	69.31	1.1	0.800000	
IPI00301579	cDNA FLJ59142, highly similar to Epididymal secretory protein E1	7.96	1	39.81	61.76	1.2	0.900000	

Differentially expressed proteins detected using stringent filtering criteria are highlighted in bold.

## Discussion

This study highlights for the first time the differential expression of AH proteins in eyes with a glaucoma shunt device compared to normal controls. Many of the identified proteins except fibronectin [30] and PEDF [31] are novel proteins that to our knowledge have not been detected in the AH of glaucoma patients. Interestingly, all of the identified proteins except C4a are known plasma proteins and increased expression in the AH suggests a breach in the blood aqueous barrier caused by a glaucoma drainage device. AH proteome changes identified in this study may not only help elucidate glaucoma pathogenesis but also shed light on the possible mechanisms that result in corneal endothelial damage and hence accelerated corneal transplant failure in eyes with glaucoma surgery.

We found a significant upregulation of complement C4a (fold change, 5; p=0.0004), an activated fragment of complement component C4. Complement activation is under the tight control of complement inhibitors; uncontrolled complement activation can cause cell lysis and inflammation while a balanced activation is necessary for clearing tissue debris and in healing. Imbalance in complement regulation has also been suggested to contribute to the neurodegenerative damage characteristic of glaucoma [32]. Activated complement in the AH, as seen in this study, could possibly cause corneal endothelial damage via direct cell lysis and inflammation.

There was evidence of enhanced fibrinolytic and coagulative activity in the AH as suggested by differential expression of prothrombin (fold change, 2.9; p=0.0004), angiotensinogen (fold change, 4.4; p=0.0001) and plasminogen (fold change, 6.2; p=0.00005). O’Brien et al. [33] have reported elevated prothrombin levels in the plasma of patients with primary open angle glaucoma and have implicated this hypercoagulable state in glaucoma pathophysiology. Angiotensinogen is a component of the renin-angiotensin (RAS) system and plays a role in the regulation of AH dynamics [34]. Plasminogen is a component of the plasmin system, and the main physiologic inhibitor of the plasmin system is plasminogen activator inhibitor-1 (PAI-1). Elevated levels of PAI-1 have been reported in the AH of glaucoma patients thus contributing to glaucoma pathogenesis by reducing proteolysis of the extracellular matrix in the TM and increasing resistance to outflow [35]. Prothrombin is also recognized as a biomarker of systemic sepsis and inflammation [36] and elevated levels in the AH suggest increased inflammation due to a breach in the blood-aqueous barrier caused by the glaucoma shunt device. Increased inflammation has been shown to stimulate increased synthesis of pro-inflammatory cytokines like interleukin-1 and tumor necrosis factor by the corneal endothelium [37,38] leading to corneal endothelial damage.

Afamin (fold change, 3.3; p=0.0003) and gelsolin (fold change, 4.7; p=0.00005) were the 2 extracellular chaperones found to be differentially expressed in the AH of patients with glaucoma shunt device compared to normals. Afamin is a member of the albumin multigene family with vitamin E-binding properties. It plays a crucial role in protecting against oxidative damage and displays neuroprotective activity not only by virtue of binding and transporting vitamin E but also on its own [39]. Gelsolin is an anti-oxidant and anti-apoptotic protein that has been implicated as a therapeutic target in Alzheimer disease since it has been shown to reduce amyloid load by inhibiting Abeta fibrillization in animal studies [40]. A decreased level of gelsolin has been observed in patients with sepsis, myocardial infarction and inflammation while an increased level has been noted in amyloidosis [41,42], so it could be a secondary response to increased amyloid load The upregulation of these extracellular chaperones is likely a response to the increased oxidative stress in the aqueous secondary to glaucoma. Oxidative stress has been recognized as the main pathogenic factor underlying open angle glaucoma [43-45]. It is likely that this may also contribute to corneal endothelial damage. Increased expression of these proteins may reflect the inability of these extracellular chaperones to completely inhibit oxidative and apoptotic damage both in the TM as well as the corneal endothelium.

Pigment epithelium-derived factor (PEDF), a member of the serpin family of proteins and expressed in all ocular tissues of the human eye, was significantly upregulated in eyes with a glaucoma shunt device (fold change, 4.6; p=0.0002). It is neuroprotective and anti-angiogenic and recently recognized as an endogenous anti-inflammatory factor [46,47]. Significantly reduced levels have been reported in advanced glaucoma AH compared to normal controls [31]. Additionally in animal models, PEDF has been shown to protect retinal ganglion cells from pressure-induced ischemia [48]. Our finding of significantly increased expression of PEDF is intriguing and leads us to speculate that perhaps this protein serves a protective role in the AH.

The function of Dickkopf-3 (Dkk3) is unclear; however, Jung et al. [49] suggest that it may acts as an anti-apoptotic molecule by decreasing intracellular levels of reactive oxygen species. Recently Nakamura et al. [50] have demonstrated that it may play a cytoprotective role in the retina by reducing caspase activity and hence protecting against apoptosis. Its role in glaucoma and corneal endothelial damage needs further evaluation.

Increased expression of apolipoprotein A-I (fold change, 6.4; p=0.0004) and A-II (fold change, 18.4; p=0.0002) was observed in this study. Apo A-1 and – II, by virtue of their association with high density lipoprotein (HDL), have anti-inflammatory properties [51]. However, its specific role in glaucoma and corneal endothelial damage is unclear and requires further investigation.

Fibronectin, an extracellular matrix glycoprotein, was increased sixfold in this study compared to normal controls (p=0.0003). This suggests disruption of the blood aqueous barrier that occurs in eyes with a glaucoma shunt device. Vesaluoma et al. [30] have demonstrated increased expression of this protein in eyes with pseudoexfoliation glaucoma. Increased fibronectin can transform TM cells and decrease the breakdown of extracellular matrix material, allowing excess to accumulate. This could ultimately reduce trabecular outflow and raise IOP.

RIG-like 7–1 constitute a family of pattern recognition receptors (PRRs). They mediate the initial sensing of microbial and endogenous danger-associated molecules that are released by tissue damage. By activating transcription of inflammatory genes they are known to control the immediate innate immune response as well as the subsequent adaptive immune response [52]. Increased levels of PRR suggests an alteration in the immunologic milieu of the AH secondary to a breach in the blood aqueous barrier.

Beta-2 microglobulin is a protein associated with major histocompatibility complex class I antigens and has value as a marker for immunologic monitoring with increased levels associated with renal and cardiac allograft rejection [53,54]. Elevated levels as seen in this study (fold change, 5.7; p=0.0002) suggest a potential role for this protein as a biomarker of increased immune mediated corneal endothelial damage in eyes with a glaucoma shunt device.

Based on the protein profile detected in this study we have hypothesized the likely mechanisms underlying corneal endothelial damage in eyes with a shunt device as well as new insights into glaucoma pathophysiology. Glaucoma per se also causes corneal endothelial damage and in the presence of glaucoma shunt device there is likely to be an exaggerated stress response leading to corneal endothelial damage and endothelial failure. This has important implications especially in the setting of corneal transplants. Corneal grafts have significantly poor long-term survival in the presence of a shunt device and future work should be targeted at identifying the specific role for these proteins so that they could potentially serve as therapeutic targets to improve graft outcomes.

This study has several strengths that need to be highlighted. The AH samples were not pooled but analyzed individually to determine proteins associated with a shunt device. We used conservative criteria for determining which proteins were differentially expressed between groups and were able to identify highly significant proteins with a large fold change compared to normals. It has been shown that the proteomic profile in glaucoma patients can vary depending on the severity of visual field defects [22]. The study patients had advanced glaucoma and this could partly explain the identification of novel proteins. The limitations of this study include the small sample size, although it is comparable to previous studies evaluating AH in eyes with glaucoma [21,22]. A useful control group would have been glaucoma patients without a shunt device who were undergoing intra-ocular surgery. A proteomic study with glaucoma as a control group is currently ongoing and should provide more insight into the pathogenic mechanism of corneal endothelial damage specific to glaucoma shunt device. This study reports on differential expression of proteins compared to normal controls but does not provide absolute quantitative data on protein levels.

Lastly, the majority of study patients had an Ahmed glaucoma shunt, which is a valved implant designed to prevent retrograde flow of fluid from the filtering bleb into the eye, so it would be interesting to determine the mechanism of increased inflammation and/or immunologic alterations seen in these eyes. Future work should be directed at evaluating the AH proteomic expression in the presence of valved and non-valved glaucoma shunts to shed light on the possible mechanisms of corneal endothelial damage with different types of shunts.

### Conclusion

We demonstrated significantly altered expression of 13 proteins in AH of eyes with a glaucoma shunt device. Many of these proteins play a role in oxidative and apoptotic damage. The findings of this study seem to suggest similar mechanisms underlying both glaucoma and corneal endothelial damage. Future work should be targeted at identifying aqueous proteins that could potentially serve as markers for corneal endothelial damage in eyes with glaucoma shunt device.
